# Is alternate rapid maxillary expansion and constriction an effective protocol in the treatment of Class III malocclusion? A systematic review

**DOI:** 10.1590/2177-6709.21.6.034-042.oar

**Published:** 2016

**Authors:** Matheus Melo Pithon, Nathalia de Lima Santos, Camila Rangel Barreto dos Santos, Felipe Carvalho Souza Baião, Murilo Costa Rangel Pinheiro, Manoel Matos, Ianderlei Andrade Souza, Rafael Pereira de Paula

**Affiliations:** 1 Professor, Department of Orthodontics, Universidade Estadual do Sudoeste da Bahia, Vitória da Conquista, Bahia, Brazil.; 2 Graduation student in Dentistry, Universidade Estadual do Sudoeste da Bahia, Vitória da Conquista, Bahia, Brazil.; 3 Professor, Dental Prosthesis Department, Universidade Estadual do Sudoeste da Bahia, Vitória da Conquista, Bahia, Brazil.; 4 Professor, Department of Endodontics, Universidade Estadual do Sudoeste da Bahia, Vitória da Conquista, Bahia, Brazil.; 5 Professor of Physical Therapy, Universidade Estadual do Sudoeste da Bahia, Vitória da Conquista, Bahia, Brazil.

**Keywords:** Malocclusion, Angle Class III, Palatal expansion technique, Early diagnosis

## Abstract

**Introduction:**

the treatment of Class III malocclusion in early age is one of the greatest challenges for orthodontists, and the establishment of more effective treatment method is a constant concern for these professionals. Thus, the objective of this systematic review is to verify the effectiveness of the therapy protocol for alternate rapid maxillary expansion and constriction (Alt-RAMEC) in the early treatment of Class III malocclusion.

**Methods:**

searches were performed in the following electronic databases: Cochrane Library, Medline (EBSCO and PubMed), SciELO, LILACS and Scopus. The following inclusion criteria were used: *in vivo* studies conducted with early intervention (patient in craniofacial development phase) with the use of the Alt-RAMEC protocol. Reviews, case reports, editorials, and studies with syndromic patients or under use of systemic drug were excluded. Duplicates were also excluded. The studies were assessed for methodological quality using the Cochrane tool for assessment of risk of bias, and classified as high or low risk of bias.

**Results:**

53 articles were found. Duplicates exclusion was thus performed and 35 articles remained. After inclusion analysis, only 5 matched the criteria. Two articles were classified as low risk of bias and three as high risk of bias. It was observed that the Alt-RAMEC enable protraction in less time and with better results, promoting greater effectiveness in the protraction treatment of Class III malocclusion.

**Conclusions:**

Although there is positive evidence of the effectiveness of early treatment with the Alt-RAMEC protocol in patients with Class III malocclusion, further studies are needed to confirm its effectiveness using long-term methodology.

## resumo

**Introdução:**

o tratamento da má oclusão de Classe III em idade precoce é um dos maiores desafios para os ortodontistas, e a descoberta de métodos de tratamento mais efetivos é uma preocupação constante para esses profissionais. Assim, o objetivo da presente revisão sistemática é verificar a eficácia do protocolo de expansão rápida da maxila e constrição alternadas (Alt-RAMEC) no tratamento precoce da má oclusão de Classe III.

**Métodos:**

foram realizadas buscas nas bases de dados eletrônicas Cochrane Library, Medline (EBSCO e PubMed), SciELO, LILACS e Scopus, obedecendo aos seguintes critérios de inclusão: estudos *in vivo* em que foi realizada intervenção precoce (pacientes em fase de desenvolvimento craniofacial) com o uso do protocolo Alt-RAMEC. Foram excluídos, além das duplicatas, os relatos de caso, revisões, editoriais e estudos com pacientes sindrômicos ou sob uso de drogas sistêmicas. Os estudos foram avaliados, quanto à qualidade metodológica, por meio da ferramenta Cochrane para Avaliação de Risco de Viés, e classificados como alto ou baixo risco de viés.

**Resultados:**

foram encontrados 53 artigos e, após removidas as duplicatas, restaram 35 artigos, dos quais 8 foram lidos na íntegra, mas apenas 5 se enquadraram nos critérios de inclusão. Desses, apenas 2 foram classificados como tendo baixo risco de viés e 3 como tendo alto risco de viés. Observou-se que o Alt-RAMEC promove a protração em menor tempo e com melhores resultados, assim como possui maior efetividade no tratamento da má oclusão de Classe III.

**Conclusões:**

apesar das evidências positivas da efetividade do tratamento precoce de pacientes com má oclusão de Classe III usando o protocolo Alt-RAMEC, ainda são necessários mais estudos em longo prazo para confirmar sua efetividade.

## INTRODUCTION

Originally defined by Angle^1^, in 1907, as a mesial relationship of molars and lower canines, the Class III malocclusion is actually linked to numerous facial features, with different skeletal and dental combinations.^2^ ^,^ ^3^ The proper diagnosis of this kind of malocclusion is indispensable for the treatment’s decision, since it is possible to observe the involvement of many tissues, such as teeth, bones, and muscles, which characterize the types of Class III malocclusions as dental, skeletal and functional, respectively.^4^ The development of Class III malocclusion can therefore include skeletal maxillary retrusion, skeletal mandibular protrusion or a combination of these two alterations.^2^ ^,^ ^3^ ^,^ ^5^

Due to the worsening of this malocclusion along the patient’s life, correct diagnosis and early treatment aim at promoting a favorable growth environment, minimizing the treatment complexity in the adult.^5^ It is known that it is during the mixed dentition period that the greatest morphological changes happen and, therefore, the proper use of intervention procedures in this period must be emphasized.^6^ ^,^ ^7^

Class III malocclusion treatment is a challenge to the orthodontist, and its approach requires greater attention in the diagnostic phase and in the decisions involving the time of treatment onset and the type of intervention to be performed. Thus, as alternative, early treatment by means of non-surgical procedures can be employed. These can be conducted by different types of protocols, providing a dynamic development process which may change its direction during the treatment, allowing some maxillary adjustments.^7^ ^,^ ^8^ A protocol to exemplify this concept is the Alt-RAMEC created by Liou and Tsai^9^ in 2005.

The Alt-RAMEC protocol confers alternate movements of rapid maxillary expansions and constrictions. The expander device used in the protocol consists of double-hinged intraoral maxillary protraction springs made of beta-titanium alloy. The main goal of this protocol is to generate the greatest maxillary expansion, allowing largest maxillary protraction, since the effectiveness of protraction depends on the opening of the circumaxillary sutures. An adequate opening of these sutures is the basic prerequisite for a good amount of maxillary protraction.^8^ ^,^ ^9^

However, this new treatment protocol for Class III malocclusion is still controversial. Thus, the objective of this systematic review is to assess by scientific evidences the effectiveness of early therapy for Class III malocclusion using maxillary protraction and disjunction, with the alternate expansion and contraction movements of Alt-RAMEC protocol.

## MATERIALS AND METHODS

### Search strategy

The review was conducted according to the PRISMA guidelines (www.prisma-statement.org). To identify relevant studies from 1900 to February 2016, a language-independent search was conducted in the following databases: Cochrane Library, Medline EBSCO and PubMed, SciELO, LILACS and Scopus. Independent searches throughout the reference lists of the retrieved articles were also conducted, in addition to the *Journal of Craniofacial Research*. The search strategy included appropriate changes in the keywords, following syntax rules of each database (Table 1).

**Table 1:** Database, method of search and number of articles retrieved.

	Search strategy	Results	Selected
Pubmed	(Alt-RAMEC OR Alternate Rapid Maxillary Expansion and Constriction) OR (Angle class III AND (Alt-RAMEC OR Alternate rapid maxillary expansion and constriction)) OR (growing Class III patients AND (alt-RAMEC OR alternate rapid maxillary expansion and constriction))	11	5
Cochrane	(Alt-RAMEC OR Alternate Rapid Maxillary Expansion and Constriction) OR (Angle Class III AND (Alt-RAMEC OR Alternate rapid maxillary expansion and constriction)) OR (growing Class III patients AND (alt-RAMEC OR alternate rapid maxillary expansion and constriction))	3	1
Medline EBSCO	(Alt-RAMEC OR Alternate Rapid Maxillary Expansion and Constriction) OR (Angle Class III AND (Alt-RAMEC OR Alternate rapid maxillary expansion and constriction)) OR (growing Class III patients AND (alt-RAMEC OR alternate rapid maxillary expansion and constriction))	11	5
SciELO	(Alt-RAMEC OR Alternate Rapid Maxillary Expansion and Constriction) OR (Angle Class III AND (Alt-RAMEC OR Alternate rapid maxillary expansion and constriction)) OR (growing Class III patients AND (alt-RAMEC OR alternate rapid maxillary expansion and constriction))	6	0
LiLacs	(Alt-RAMEC OR Alternate Rapid Maxillary Expansion and Constriction) OR (Angle Class III AND (Alt-RAMEC OR Alternate rapid maxillary expansion and constriction)) OR (growing Class III patients AND (alt-RAMEC OR alternate rapid maxillary expansion and constriction))	7	0
Scopus	(TITLE-ABS-KEY (alt-RAMEC OR alternate rapid maxillary expansion AND constriction ) OR TITLE-ABS-KEY (Angle Class III AND (alt-RAMEC OR alternate rapid maxillary expansion AND constriction)) OR TITLE-ABS-KEY (growing Class III patients AND (alt-RAMEC OR alternate rapid maxillary expansion AND constriction)))	10	3
Electronic Journal and manual search	(Alt-RAMEC OR Alternate Rapid Maxillary Expansion and Constriction) OR (Angle Class III AND (Alt-RAMEC OR Alternate rapid maxillary expansion and constriction)) OR (growing Class III patients AND (alt-RAMEC OR alternate rapid maxillary expansion and constriction))	5	3
Total articles retrieved	53	17	
Total without repetitions	35	6	

The articles were selected based on title and abstract, and had to match the following inclusion criteria: controlled clinical studies with patients in the growth phase (during craniofacial development) with Class III malocclusion (P-participants), who were submitted to the Alt-RAMEC protocol for maxillary expansion and protraction as early treatment (I-intervention). The articles also had to compare individuals of same gender and age, and also with the traditional method of maxillary expansion (C-comparison), establishing from the results whether there was a greater effectiveness of Alt-RAMEC or not (O-outcome) (Table 2). Were excluded: case reports, review articles, editorial or personal opinions, patients using systemic medications and/or with systemic disorders, and patients submitted to previous surgical procedures involving maxilla and/or mandible.

**Table 2:** Inclusion criteria based on the PICO format.

	Inclusion criteria
P (participants)	Individuals in the growing phase and with anteroposterior and transverse maxillary deficiency.
I (intervention)	Use of the Alt-RAMEC protocol for maxillary expansion and protraction.
C (comparison)	Individuals of same age and sex, and treated with traditional method of maxillary expansion.
O (outcomes)	Hypothesis: greater effectiveness in the maxillary expansion and protraction with Alt-RAMEC protocol than with traditional methods. Null hypothesis: there is no difference between Alt-RAMEC protocol and traditional methods for maxillary expansion and protraction.

The initial analysis excluded articles with titles and abstracts not related to the studied issue and presenting at least one of the exclusion criteria. The next step was a detailed analysis of selected articles, to examine those who respected all inclusion criteria or presented exclusion criteria. When the information in the title or abstract was insufficient to decide about the inclusion or exclusion, the full article was read and then decided about its inclusion or exclusion. Articles without abstract were read entirely to define their eligibility.

Article selection was performed by two researchers based on critical analyses regarding the inclusion and exclusion criteria. If discrepancies were found between the two researchers, a new reviewer was added, to eliminate the discrepancies between the other two evaluators. When consensus was obtained among the reviewers regarding the articles that met the inclusion criteria, these were finally included in the systematic review. In cases where additional data were needed, authors were contacted via email to obtain these additional information.

### Quality assessment and risk of bias

To assess the methodological quality and risk of bias of the included studies, it was used the The Cochrane Collaboration’s Tool for Assessing Risk of Bias, published in the Cochrane Handbook for Systematic Reviews of Interventions (Version 5.1.0).^10^

The selection bias consists in the systematic difference between the baseline group and the other groups, composed by the domains of sequence generation and allocation concealment. The performance bias comprises systematic differences between the groups in which the care is provided, or submitted to other factors than the interventions of interest, including the domains of blinding of participants and staff and other potential threats to validity.

The detection bias was assessed by the blinding of outcome assessment, which consists of systematic differences of how are defined the results between the groups. The attrition bias was evaluated by the domain of incomplete outcome data, comprising systematic differences between the groups that were drawn from the study. And the reporting bias includes the systematic differences between the reported and unreported results, being evaluated by the domains of selective reporting. 

For each assessed domain the articles could be classified as low risk (green circles in the Figure 2), high risk (red circles), or unclear, if not enough information was given to allow an adequate classification (Fig 2).

**Figure 1:**
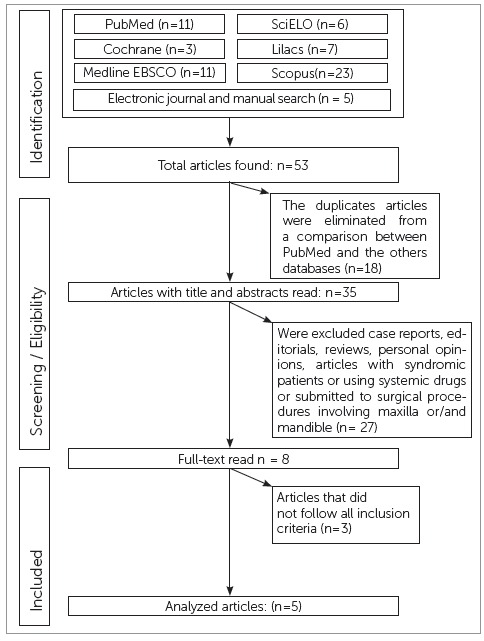
Flow chart: results of searches.

**Figure 2:**
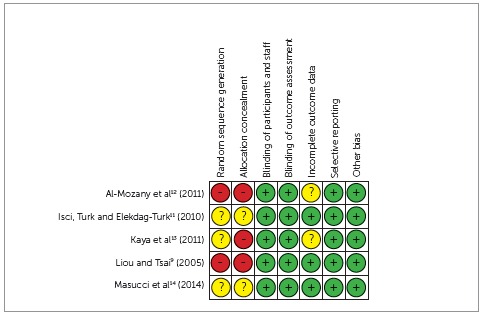
Quality assessment of the selected studies (The Cochrane Collaboration Tool For Assessing Risk of Bias).

## RESULTS

During the first step of selection and evaluation process, 53 articles were selected -based on the abstracts and/or titles- from PubMed database, and a comparison was made with other databases to eliminate duplicated studies. As a result, 35 articles were retrieved and the ones that did not fulfill the inclusion criteria were excluded. Then 8 articles remained, which were read completely. Finally, 5 articles were included in this systematic review as shown in the flowchart (Fig 1).

After submission of the articles to the assessment tool for risk of bias, 2 studies were classified as low risk of bias^11^ ^,^ ^12^ and the other 3 articles as of high risk of bias,^9^ ^,^ ^13^ ^,^ ^14^ as illustrated in Figure 2. 

With regard to the sequence of random generation, two studies did not conduct it,^9^ ^,^ ^11^ the other did not provide sufficient information for trial in that domain. As to the allocation of participants in all groups were considered as high risk of bias, due to the way that the allocation occurred or for having only one group.

 Due to the use of different devices and orthodontic treatments, the blinding of participants and staff was not possible because they are easily distinguishable. However, it does not assign to a high risk of bias. As for the blinding corresponding to the statistical evaluation, all articles were classified as low risk of bias.

In relation to incomplete data in the results, two studies did not provide enough information to allow the respective ranking, coinciding with the studies that did not have a control group.^12^ ^,^ ^13^ For the evaluation of selective reporting all studies were considered as low risk of bias.

Table 3 shows the data extracted from the articles: author, year, sample, mean age, inclusion criteria, treatment, outcomes and p-value. The age of the participants ranged from 8 to 12 years old. The sample size of the studies ranged from 14 to 31 participants. Two studies compared the Alt-RAMEC protocol with the Rapid Maxillary Expansion (RME),^9^ ^,^ ^11^ ^,^ ^14^ and one of those has a control group without intervention. Two studies assessed only the Alt-RAMEC protocol:^12^ ^,^ ^13^ Kaya et al^13^ used the facemask, while Al-Mozany^12^ used skeletal anchorage devices.

**Table 3:** Description of included studies.

	Participants			Intervention			
Author	Total	Average age	Conditions	Type of treatment	Results	p-value
Liou, Tsai^9^ (2005)	RME group: 16 (8F/8M) Alt-RAMEC group: 10 (6F/4M)	9 to 12 years	Patients with cleft lip palate in mixed dentition	» Rapid maxillary expansion (RME) for 1 week followed by 5 months, 3 weeks of maxillary protraction. - The Alternate Rapid Maxillary Expansions and Constrictions for 9 weeks followed by 3 months, 3 weeks of maxillary protraction.	Alt-RAMEC protocol caused a maxillary protraction in a shorter period of time, with better results, promoting a jaw displacement almost double with relation to the RME group, without significant relapse for a period of 2 years after treatment.	*p* < 0.05
Isci et al^11^ (2010)	RPE group: 15 (8F/7M) A/D-RPE group: 15 (8F/7M)	RPE group: 11.94 ± 1.62 years A/D-RPE group: 11.34 ± 1.81 years	Patients with Class III malocclusion compared; negative overjet; with erupted first-premolars; concave profile and skeletal Class III pattern due to retruded maxilla, with or without mandibular protrusion.	» Rapid palatal expansion (RPE) for 1 week. » Activation and deactivation (A/D) RPE protocol with reverse headgear (RH).	Alternately repeating the protocol (Alt-RAMEC) with HR had greater effectiveness with respect to jaw movement when compared to the other group, with approximately twice the movement, and no significant relapses were observed.	*p* < 0.001
Al-Mozany et al^12^ (2011)	14 (7F/ 7M)	11.02 ± 14.02 years	Patients on the waiting list at the University of Sydney, who had Class III malocclusion with maxillary retrognathism, in a state of cervical maturation.	Alt-RAMEC protocol with skeletal anchorage device (TAD)	Using the Alt-RAMEC protocol with TAD’s demonstrated efficiency, as discarded the possibility of using external devices for maxillary protraction.	*p* < 0.001
Kaya et al^13^ (2011)	15 (9F/6M)	11.6 ± 1.59 years	No history of previous orthodontic/orthopedic treatment, no systemic diseases or congenital deformities, concave profile, skeletal and dental Class III malocclusion, edge-to-edge/reverse incisor relationship, and symptom-free temporomandibular joint function.	Alt-RAMEC protocol for 8 weeks followed by maxillary protraction using facemask	Significant forward movement of the maxilla and clockwise rotation of the mandible, with a slight counterclockwise rotation and without maxillary incisor proclination and slight uprighting of the mandibular incisors, respectively. Statistically significant increase in vertical dimension and improvement in soft tissue profile.	*p* < 0.001
Masucci et al^14^ (2011)	Alt-RAMEC group: 31 (14F/17M) RME/FM group: 31 (15F/ 16M) Control group: 21 (12F/9M)	Alt-RAMEC: 8.1 ± 0.9 years RME/FM: 8.5 ± 1.3 years Control: 8.0 ± 1.1 years	European ancestry, anterior crossbite or edge-to-edge incisor relationship, accentuated mesial step relationships of the primary second molars or Class III relationships of the permanent first molars, Wits appraisal ≤ 2.0 mm, absence of CO-CR discrepancy, deciduous or early mixed phase of dentition, pre-pubertal skeletal maturation (CS1 to CS2).	» Modified alternate rapid maxillary expansions and constrictions (Alt-RAMEC) protocol with facemask (FM) » Rapid maxillary expansion and facemask (RME/FM) » Control group without treatment	The modified Alt-RAMEC/FM protocol allows obtaining more favorable skeletal effects in terms of maxillary advancement, leading to a greater improvement in sagittal skeletal relationships as compared to the conventional RME/FM protocol. Both groups showed similar effects as for mandibular skeletal changes and vertical skeletal relationships.	*p* < 0.001

Regarding the results of the articles included in this systematic review, Isci, Turk and Elekdag-Turk^11^ showed greater effectiveness in the group that used the alternate repetitive protocol (Alt-RAMEC) associated to HR. The Alt-RAMEC group exhibited approximately a two-fold magnitude of maxillary movement, when compared to the other group. Al-Mozany^12^ used Temporary Anchorage Devices (TADs) and Class III intermaxillary elastics, associated with the Alt-RAMEC protocol to correct Class III malocclusions. According to the authors, this method has shown to be effective to treat patients with maxillary deficiency, eliminating the need of external maxillary protraction devices. However, the authors pointed out for the need of studies aiming to assess the long-term stability.

Liou and Tsai^9^ found that Alt-RAMEC protocol caused a maxillary protraction in a shorter period of time, with better results, promoting a jaw displacement almost double with relation to the RME group. As regard, the modified Alt-RAMEC/FM protocol allows obtaining more favorable skeletal effects in terms of maxillary advancement, leading to a greater improvement in sagittal skeletal relationships as compared to the conventional RME/FM protocol.^14^ Both groups showed similar effects for mandibular skeletal changes and vertical skeletal relationships. Kaya et al^13^ found statistically significant increase in vertical dimension and improvement in soft tissue profile.

## DISCUSSION

Rapid maxillary expansion procedures have been proposed since the last century by Angell and clinically consolidated by Haas in 1961.^15^ These procedures lead to an increase in the transverse dimensions of the upper arch by skeletal changes, associated with dental abnormalities, which may manifest in distinct ways, depending on resistance of the sutures, which increases as a person matures.^16^ ^,^ ^17^ ^,^ ^18^

An alternative to disarticulate the circumaxillary sutures without excessive maxillary expansion was proposed by Liou and Tsai^9^ in 2005 (Alt-RAMEC protocol). This new system confers alternate rapid maxillary expansions and contractions, aiming to disarticulate the surrounding sutures to treat cases of Class III malocclusion in patients in the growing phase.^9^ Although widespread in the orthodontic literature in the recent years, this protocol requires scientific evidence.

Based on the aforementioned, this systematic review was proposed to seek evidence about the effectiveness and stability of the Alt-RAMEC protocol, when used in the early treatment of Class III malocclusion, compared to other methods for rapid maxillary expansion. Thus, only studies using this new protocol were included, whenever it was the original Alt-RAMEC protocol (i.e., using alternate rapid maxillary expansions and contractions) or the Alt-RAMEC protocol followed by another method of maxillary traction. It is noteworthy that meta-analysis was tried, but it was not possible to be performed due to the high degree of heterogeneity between the studies.

Comparative studies developed by Liou and Tsai^9^ reported that a repetitive weekly protocol using Alt-RAMEC with bi-articulated expander displaces the maxilla more anteriorly and disarticulates the circumaxillary sutures more effectively than conventional maxillary expansion devices, resulting in better effectiveness of maxillary protraction. According to the authors, the magnitude of anterior maxillary displacement in the Alt-RAMEC group was almost two times higher than in the RME group. Both groups used bi-articulated expanders, but with different protocols. The intraoral protraction springs on both groups had a similar force magnitude. However, the protraction in the Alt-RAMEC group was 8 weeks faster than in the RME group. It seems evident that the greater amount of maxillary advancement in Alt-RAMEC group was related to repetitive weekly Alt-RAMEC protocol. Thus, the authors highlight that the repetitive weekly protocol seems to be more influent than the type of expander used.

### Strength

Regarding quantification of the force applied in the treatment with Alt-RAMEC, Liou and Tsai^9^ and Al-Mozany^12^ reported the use of force around 400 g. Isci, Turk and Elekdag-Turk^11^ reported a force of 700 g with the Reverse Headgear (RH) used after the active period of the protocol. It can therefore be inferred that there is an agreement in 400 g of force application. Kaya et al^13^ used 100 g of force per side, applied via elastic between the miniplates and facemask. The force was increased by 350-400g per side during the second week of treatment. Masucci et al^14^ used force ranging between 400 and 500g.

### Activation period

As regards to the activation periods, Liou and Tsai^9^ postulated for a 1 mm activation per day for each week (expansion and constriction) till the completion of 9 weeks. The same protocol was used by Al-Mozany et al.^12^ The study with a modification of this standard was proposed by Isci, Turk and Elekdag-Turk,^11^ who used activation twice a day, and 0.20 mm for each shift for a week (alternating expansion and constriction), up to 4 weeks. Thereafter, 16-18h RH for 3 months was performed, followed by another 3 months with 12h/day, ending with 6 months of 6h daily use. A similarity in the device activation processes can be observed, with predominance of daily activation for one week (1 mm) for extension and another week for constriction. Kaya et al^13^ used the screw of the RME appliance alternately opened and closed for 2-week periods over the course of 8 weeks. The treatment protocol began with expansion, followed by final constriction. Daily activation for the expansion/constriction course was 0.5 mm. Masucci et al^14^ used a protocol which was activated by the patient’s parents twice a day (0.20 mm per turn, one turn in the morning and one turn at night) for 1 week, then it was deactivated twice a day (one turn in the morning and one turn at night) for 1 week. This alternating protocol was repeated twice. After 4 weeks of Alt-RAMEC therapy, an additional twice-daily activation of the expansion screw was performed until overcorrection was achieved. At the end of the expansion phase, a face mask was placed for maxillary protraction.

### Follow-up

In regard to the follow-up period, only the study of Liou and Tsai^9^ points out a two years follow-up without recurrence. Isci, Turk and Elekdag-Turk^11^ claims not to having occurred reappearance, yet the period of post-treatment follow-up is not explicit. Al-Mozany et al^12^ did not informed follow-up. Thus, we perceive the need of studies that follow participants up for a longer period, allowing to analyze the stability of the treatment over the years.

Using a modified Alt-RAMEC protocol, without the use of intraoral springs, Isci, Turk and Elekdag-Turk^11^ conducted a comparative study with 30 patients, comparing two types of maxillary expansion. One group was submitted to a rapid maxillary expansion (RME) and another to a maxillary expansion with activation-deactivation of Alt-RAMEC (A/D-RME), both protocols with reverse headgear (RH). In the final analysis, there was a significant difference in the correction of overjet between the two groups. Correction of overjet was greater in the group treated with maxillary expansion using the modified Alt-RAMEC protocol (i.e., A/D-RME and HR) due to a greater forward movement of the maxilla and maxillary incisors; an improvement of the soft tissue profile was also observed. These positive results were maintained without significant relapses.

Isci, Turk and Elekdag-Turk^11^; Al-Mozany et al.^12^ introduced TADs together with Class III intermaxillary elastics to produce movement protraction, aiming to eliminate the need for extra-oral devices. Thus, the authors joined the TADs and Alt-RAMEC protocol to evaluate the effectiveness of the use of TADs and Class III intermaxillary elastics in patients with maxillary retrognathia, in skeletal growth, and whose maxilla was previously disarticulated by the Alt-RAMEC protocol. The combination of Alt-RAMEC protocol for maxillary disarticulation and TADs in the maxilla and mandible together with Class III intermaxillary elastic proved to be an effective and well-tolerated method for the treatment of patients with Class III malocclusion. This treatment protocol achieved significant skeletal protraction of the maxilla in a considerably reduced time. However, the long-term stability of these changes need to be evaluated.^12^

Although a study was conducted with patients with cleft lip and palate, it did not detect any difference in treatment in non cleft patients, with exception to the making of the piece that was modeled so that the jackscrew stayed oriented perpendicularly to the alveolar fissure.

Analyzing the results from the Alt-RAMEC protocol associated to TADs, the study pointed the effectiveness to treat Class III malocclusion patients. On the other hand, the authors postulate that the Alt-RAMEC alone does not increase the amount of maxillary forward movement, thus other factors such as age of the patient, duration of the mask use and treatment exposure should be considered. Therefore, further studies are needed to clarify the stability of this protocol.

It may be seen throughout this review that all selected studies comparing the use of Alt-RAMEC and other traditional expansion protocols concluded that there are significantly different results, with better results for the group treated with the Alt-RAMEC protocol. However, there is a gap in the literature, since there were no studies focusing on assessing the stability of the results on the long-term (over two years). Further studies, with long-term design, are necessary to evaluate the stability maintenance, which is important for Class III malocclusion treatment protocols.

## CONCLUSION

From the analysis of the articles, it was concluded that:

» The use of Alt-RAMEC protocol is effective to early treatment of Class III malocclusion patients.
» The stability of the correction of Class III malocclusion could not be verified, because of the lack of studies designed to assess this issue.
» Although the scientific evidence point to a greater effectiveness of the protocol using Alt-RAMEC in the early treatment of Class III malocclusion, more studies are needed with longer follow-up period, as well as better definition of the test and control groups.
